# Supplementary material for: Coverage with Timely Administered Vaccination against Hepatitis B Virus and Its Influence on the Prevalence of HBV Infection in the Regions of Different Endemicity
Source: Vaccines (Basel). 2021 Jan 23;9(2):82. doi: 10.3390/vaccines9020082 (PMC7912110; doi:10.3390/vaccines9020082)
Supplement: Supplementary file 1 [file vaccines-09-00082-s001.zip › Supplementary Figure S1.pdf]

SURVEY ON ATTITUDE TO VACCINATION

INTERVIEWER, READ OUT: " Hello, my name is ___________. I conduct a survey on attitude towards vaccination.

SECTION 1. RESPONDENT SELECTION

Do you agree to participate in the survey?

1	Yes	2	No => *Stop the interview*

SECTION 2. TECHNICAL INFORMATION

2.1 Interviewer ID: |_____|_____|_____|_____|

2.2 Date of interview, Day: |_____|_____| Month: |_____|_____|
2.3 Interview start time, Hours: |_____|_____| Minutes: |_____|_____|
2.4 Institution name: ____
2.5 Place: ____

SECTION 3. RESPONDENT INFORMATION

INTERVIEWER, READ OUT: First, I will ask you a few questions about you.

3.1 *INTERVIEWER, INDICATE THE GENDER OF THE RESPONDENT*

1	Male	2	Female

3.2 How old are you? ________

3.3. What education do you have? *INTERVIEWER, SPECIFY STUDENT OR TEACHER*

1	Student	2	Teacher

SECTION 4. KNOWLEDGE OF VACCINATION

4.1 Please name the ways to prevent dangerous infections such as tuberculosis, hepatitis, diphtheria, measles, etc.? *(respondents were not prompted with a list of possible answers)*

1	Compliance with the rules of hygiene
2	Physical activity and tempering
3	Healthy food, quality of drinking water
4	Vaccination
5	Immunomodulators
6	Vitamins/dietary supplements
7	There are no such ways
8	Other (INDICATE)

4.2. Do you know what vaccination is?

1	Yes	2	No

INTERVIEWER, IF RESPONDENT ANSWERED NO, EXPLAIN THAT Vaccination is the use of prophylactic immunizations in order to induce immunity to the disease, which will prevent infection or reduce its negative effects.

4.3 What infections do you think can be prevented by vaccination? *(respondents were not prompted with a list of possible answers)*

4.4 *INTERVIEWER, ASK A QUESTION ABOUT ALL INFECTIONS NOT MENTIONED BY RESPONDENT*. **Have you heard that** */INFECTION NAME/* **can be prevented by vaccination?**

	**Variant 4.4 (a)**<br>Spontaneous mention	**Variant 4.4 (b)**<br>Mention with hint
Diphtheria		
Measles		
Tetanus		
Hepatitis A		
Hepatitis B		
Mumps		
Poliomyelitis		
Influenza		
Rubella		
Rabies		
Chickenpox		
Human papillomavirus		
Tick-borne encephalitis		
Yellow fever		
Meningococcal infection		

SECTION 5. ATTITUDE TOWARDS THE VACCINATION

5.1 Heard about the National Vaccination Schedule

1	Yes	2	No

5.2. Vaccination fears *(multiple answer choices, a list of possible answers was read out)*

1	Have no fears about vaccination
2	Children receive too many vaccines in the first two years of life
3	Vaccines can cause side effects or complications
4	Vaccines can weaken the immune system of children and adults
5	Vaccines can cause disease
6	The ingredients in vaccines are unsafe
7	The long-term effects of vaccines on humans have not been studied
8	Vaccination is against my religious beliefs
9	The quality of vaccines is low
10	Other

5.3. Have you or your relatives/acquaintances encountered illness/medical complications in children caused by vaccination?

1	Yes	2	No

5.4 Have you or your relatives/acquaintances encountered serious illness that could have been prevented by vaccination?

1	Yes	2	No

5.5 There are people/groups of people who refuse to vaccinate themselves and their children. How do you feel about their decision? *(Choose one answer from the list)*

1	I disagree. They risk the health of their children and contribute to the spread of disease
2	I do not care. They are entitled to their opinion
3	I agree with them
4	I haven't heard anything about this
5	Other

5.6 Do you trust healthcare providers who vaccinate? *(Choose one answer from the list)*

1	I completely trust them
2	I mostly trust them
3	No opinion
4	I mostly do not trust them
5	I do not trust them at all

5.7 Do you trust the quality of vaccines? *(Choose one answer from the list)*

1	I completely trust the quality of vaccines
2	I mostly trust the quality of vaccines
3	No opinion
4	I mostly do not trust the quality of vaccines
5	I do not trust the quality of vaccines at all

5.8 What information about vaccinations would you like to receive? *(Choose any answers from the list)*

1	Information about the usefulness and benefits of vaccination
2	Information about additional vaccines
3	Information about vaccine composition
4	Information about contraindications to vaccination
5	Information about complications after vaccination
6	Information about the manufacturers of vaccines
7	Other
8	I have enough information

INTERVIEWER, PLEASE THANK THE RESPONDENT
