# Supplementary material for: Coverage with Timely Administered Vaccination against Hepatitis B Virus and Its Influence on the Prevalence of HBV Infection in the Regions of Different Endemicity
Source: Vaccines (Basel). 2021 Jan 23;9(2):82. doi: 10.3390/vaccines9020082 (PMC7912110; doi:10.3390/vaccines9020082)
Supplement: Supplementary file 1 [file vaccines-09-00082-s001.zip › Supplementary Table S1.docx]

Table 1. Frequency of detection of HBV markers in age cohorts of conditionally healthy population in Belgorod Oblast and Yakutia

| Region | Age, years | N tested | HBsAg  positive,  N (% [95%CI]) | Anti-HBc  positive,  N (% [95%CI]) | Anti-HBs positive,  Anti-HBc negative  N (% [95%CI]) | Anti-HBs negative,  Anti-HBc negative  N (% [95%CI]) |
| --- | --- | --- | --- | --- | --- | --- |
| Belgorod Oblast | 0-9 | 90 | 0 (0%) | 14 (15.6% [9.4-24.6%]) | 52 (57.8% [47.5-67.5%]) | 24 (26.7% [18.6-36.7%]) |
|  | 10-14 | 148 | 1 (0.7% [0.01-4.1%]) | 13 (8.8% [5.1-14.6%]) | 99 (66.9% [59.0-74.0%]) | 36 (24.3% [18.1-31.9%]) |
|  | 15-19 | 243 | 0 (0%) | 25 (10.3% [7.0-14.8%]) | 153 (63.0% [56.7-68.8%]) | 65 (26.7% [21.6-32.7%]) |
|  | 20-29 | 231 | 0 (0%) | 17 (7.4% [4.6-11.5%]) | 164 (71.0% [64.8-76.5%]) | 50 (21.6% [16.8-27.4%]) |
|  | 30-39 | 253 | 0 (0%) | 12 (4.7% [2.7-8.2%]) | 122 (48.2% [42.1-54.4%]) | 119 (47.0% [40.1-53.2%]) |
|  | 40-49 | 244 | 1 (0.4% [0.01-2.52%]) | 34 (13.9% [10.1-18.9%]) | 60 (24.6% [19.6-30.4%]) | 150 (61.5% [55.2-67.4%]) |
|  | 50-59 | 163 | 1 (0.6% [0.01-3.74%]) | 35 (21.5% [15.8-28.4%]) | 36 (22.1% [16.4-29.1%]) | 92 (56.4% [48.8-63.8%]) |
|  | ≥ 60 | 382 | 6 (1.6% [0.6-3.5%]) | 150 (39.3% [34.5-44.3%]) | 24 (6.3% [4.2-9.2%]) | 208 (54.4% [49.4-59.4%]) |
|  | Total | 1754 | 9 (0.5% [0.3-1.0%]) | 300 (17.1% [15.4-18.9%]) | 710 (40.5% [38.2-42.8%]) | 744 (42.4% [40.1-44.7%]) |
| Yakutia | 0-9 | 277 | 9 (3.3% [1.6-6.2%]) | 29 (10.5% [7.4-14.7%]) | 156 (56.3% [50.4-62.0%]) | 92 (33.2% [27.9-39.0%]) |
|  | 10-14 | 125 | 5 (4.0% [1.5-9.3%]) | 13 (10.4% [6.1-17.1%]) | 77 (61.6% [52.8-69.7%]) | 35 (28% [20.1-36.5%]) |
|  | 15-19 | 99 | 2 (2.0% [0.1-7.5%]) | 18 (18.2% [11.7-27.0%]) | 46 (46.5% [37.0-56.2%]) | 35 (35.3% [26.6-45.2%]) |
|  | 20-29 | 117 | 1 (0.9% [0.01-5.2%]) | 26 (22.2% [15.6-30.6%]) | 59 (50.4% [41.5-59.3%]) | 32 (27.3% [20.1-36.1%]) |
|  | 30-39 | 119 | 2 (1.7% [0.1-6.3%]) | 55 (46.2% [37.5-55.2%]) | 45 (37.8% [29.6-46.8%]) | 19 (16.0% [10.4-23.7%]) |
|  | 40-49 | 101 | 1 (1.0% [0.01-5.9%]) | 48 (47.5% [38.1-57.2%]) | 36 (35.6% [27.0-45.4%]) | 17 (16.8% [10.7-25.4%]) |
|  | 50-59 | 116 | 2 (1.7% [0.1-6.5%]) | 63 (54.3% [45.3-63.1%]) | 34 (29.3% [21.8-38.2%]) | 19 (16.4% [10.7-24.3%]) |
|  | ≥ 60 | 118 | 2 (1.7% [0.1-6.4%]) | 63 (53.4% [44.4-62.1%]) | 37 (31.4% [23.7-40.2%]) | 18 (15.2% [9.8-22.9%]) |
|  | Total | 1072 | 24 (2.2% [1.5-3.3%]) | 315 (29.4% [26.7-32.2%]) | 412 (38.4% [35.6-41.4%]) | 345 (69.4% [66.6-72.1%]) |
